# Computational Identification of Immune- and Ferroptosis-Related LncRNA Signature for Prognosis of Hepatocellular Carcinoma

**DOI:** 10.3389/fmolb.2021.759173

**Published:** 2021-11-25

**Authors:** Anmin Huang, Ting Li, Xueting Xie, Jinglin Xia

**Affiliations:** ^1^ Key Laboratory of Diagnosis and Treatment of Severe Hepato-Pancreatic Diseases of Zhejiang Province, The First Affiliated Hospital of Wenzhou Medical University, Wenzhou, China; ^2^ School of the First Clinical Medical Sciences, Wenzhou Medical University, Wenzhou, China; ^3^ Liver Cancer Institute, Zhongshan Hospital of Fudan University, Shanghai, China

**Keywords:** ferroptosis, immune, long non-coding RNA (lncRNA), hepatocellular carcinoma, prognosis

## Abstract

Long non-coding RNAs (lncRNAs), which were implicated in many pathophysiological processes including cancer, were frequently dysregulated in hepatocellular carcinoma (HCC). Studies have demonstrated that ferroptosis and immunity can regulate the biological behaviors of tumors. Therefore, biomarkers that combined ferroptosis, immunity, and lncRNA can be a promising candidate bioindicator in clinical therapy of cancers. Many bioinformatics methods, including Pearson correlation analysis, univariate Cox proportional hazard regression analysis, least absolute shrinkage and selection operator (LASSO) analysis, and multivariate Cox proportional hazard regression analysis were applied to develop a prognostic risk signature of immune- and ferroptosis-related lncRNA (IFLSig). Finally, eight immune- and ferroptosis-related lncRNAs (IFLncRNA) were identified to develop and IFLSig of HCC patients. We found the prognosis of patients with high IFLSig will be worse, while the prognosis of patients with low IFLSig will be better. The results provide an efficient method of uniting critical clinical information with immunological characteristics, enabling estimation of the overall survival (OS). Such an integrative prognostic model with high predictive power would have a notable impact and utility in prognosis prediction and individualized treatment strategies.

## Introduction

As one of the top five leading causes of cancer-related deaths worldwide and the fifth most frequent in China, liver cancer still has an estimated number of 392,868 new cases and 368,960 cancer-related deaths in 2018 (Feng et al., 2019). The symptoms of HCC are not obvious in the early stage, but are perceptible in the late stage, which can easily lead to delayed diagnosis and poor treatment effects (Bray et al., 2018). Therefore, individual treatment and prognosis determination of liver cancer are still major challenges.

LncRNA with transcripts longer than 200 nt is initially considered to be a meaningless plate in transcription (Chew et al., 2018). With the large-scale use of massively parallel signature sequencing technology, lncRNAs instead have shown a high correlation with liver cancer, mainly in their regulatory roles during liver carcinogenesis (Wang et al., 2015; Wang et al., 2016). These cancer-related lncRNAs participate in numerous biological processes, including epigenetic regulation, signal transduction, and cell cycle control (Yuan et al., 2017; Wu et al., 2019; Xu et al., 2019).

Ferroptosis is a recently recognized cell death modality that is intimately associated with the development of tumor-immunosuppressive microenvironment, and the existence of ferroptosis-related intercourse was evidenced between tumor cells and immune cells (Jiang et al., 2021; Xu et al., 2021). A plethora of research on the relationship between ferroptosis and immunity have given a new outlook on our understanding of the pathogenesis of liver cancer, and the intervention of ferroptosis can effectively improve immunosuppression (Gao et al., 2015; Ou et al., 2016; Sun et al., 2016; Wang et al., 2021). In the tumor microenvironment, ferroptosis can be regulated by many molecular factors, and abundant protein interactions also participate in this progress. Ferroptosis can expose the antigens in tumor cells, thereby increasing the immunogenicity of the tumor immune microenvironment and improving the availability of immunotherapy (Tang et al., 2020). In addition, several factors such as the tumor microenvironment, tumor immune-infiltrating cells, and response to immunotherapy provide a certain association with the clinical outcome of cancer (Vitale et al., 2019; Zheng et al., 2020). These theories are the cornerstone of tumor immunotherapy in the future.

A novel lncRNA prognostic risk model, combining ferroptosis and immunity, was constructed for clinical practice in this study. First of all, to provide a better strategy to orchestrate the immune system in eradicating cancers, we comprehensively analyzed the lncRNA profiles, combining ferroptosis and immunity profiles, as well as determined their clinical significance to construct a prognostic algorithm. Following this, we provide evidence of the scale of immune profile and the potential effectiveness of immunotherapy use in our prognostic risk model. We conclude with suggestions for providing an opportunity to further optimize the modeling tool for the prognosis and therapy responses of HCC.

## Materials and Methods

### Data Collection

The RNA sequencing (RNA-seq) data and corresponding clinical information of 421 cases in total (including 363 cancer samples and 58 normal samples) were obtained from The Cancer Genome Atlas database (TCGA, http://portal.gdc.cancer.gov/), and the missing and abnormal values were processed with multiple imputation by utilizing SPSS ver. 25.0 (IBM United States). Ferroptosis-related genes were obtained from the FerrDb database (http://www.zhounan.org/ferrdb/), and immune-related genes were obtained from the ImmPort database (https://www.immport.org).

### The Construction and Validation of Prognostic Risk Model

Differential expression of lncRNA genes (DEGs) was performed between cancer samples and normal samples with R package “Dseq2,” and the cut-off was set at log2foldchange (logFC) <−2, *p*-value <0.05, and adjusted *p*-value <0.01. The relevance between DEGs and ferroptosis-related genes and immune-related genes was explored by Pearson correlation analysis. Then, univariate Cox proportional regression analysis and least absolute shrinkage and selection operator (LASSO) Cox proportional regression were performed to identify candidate immune- and ferroptosis-related lncRNAs (IFLncRNA). A total of 363 HCC patients were randomized into the training cohort or test cohort for the construction and validation of risk score at the ratio of 6:4 (218 in the training cohort and 145 in the test cohort). For the training cohort, our prognostic risk signature of immune- and ferroptosis-related lncRNA (IFLSig) was structured by applying the linear combination of the expression values of each prognostic IFLncRNA, weighted by their estimated regression coefficients through the multivariate Cox proportional hazard regression analysis as follows:

Risk score(patients)=∑coefficient(lncRNA)∗expression(lncRNA)

Risk score (patients) is a latent prediction risk score for patients, and IFLSig is a prediction risk score based on IFLncRNA. The expression (lncRNA) is the expression value of IFLncRNA of HCC. The meaning of coefficient (lncRNA) is the contribution of IFLncRNA that was derived from the coefficient of multivariate Cox regression in the training cohort. The median of IFLSig in the training cohort and test cohort was used as a cut-off to sort patients into the high-risk group and the low-risk group. The Kaplan–Meier (K-M) survival curves and receiver operating characteristic (ROC) curves were used to estimate the predictive power of overall survival (OS). A principal component analysis (PCA) was applied to estimate the expression difference of IFLncRNA in HCC patients. Univariate Cox proportional hazard regression and multivariate Cox proportional hazard regression analyses for OS were performed on the independent prognostic factor of the risk score. Moreover, a nomogram was constructed with IFLSig and other clinical factors.

### Functional Enrichment Analysis

Gene Set Enrichment Analysis (GSEA) (ver. 4.1.0, http://www.gsea-msigdb.org/gsea/index.jsp) was performed to identify signal pathways and functional categories associated with IFLSig in the enrichment of Kyoto Encyclopedia of Genes and Genomes (KEGG) and Gene Ontology (GO).

### Clinical Therapy Response With IFLSig for HCC Patients

To estimate the prediction accuracy of IFLSig with immunotherapy response for clinical practice, the algorithm from CIBERSORT (https://cibersort.stanford.edu/) was used to estimate the profile of immune cell subtypes in HCC patients. The correlation between IFLSig and immune checkpoints was analyzed to confirm the potential of IFLSig as a biomarker for immune checkpoint inhibitor (ICI) treatments. The sensitivity and resistance chemotherapeutic drugs were identified by using the R package “pRRophetic.”

### Statistical Analysis

All statistical analyses were applied by R software ver. 3.6.3 and SPSS ver. 25.0. Pearson correlation analysis was performed with R package “stats.” Univariate Cox proportional hazard regression analysis, multivariate Cox proportional hazard regression analysis, and nomogram were performed with R package “survival” and “survminer.” A log-rank test was used for calculating the statistical difference in OS between the low-risk group and high-risk group. LASSO Cox proportional regression was performed with R package “glmnet.” ROC curve was performed with R package “survivalROC.” Harrell’s index of concordance (*C*-index) was performed to estimate the prognostic power of the nomogram.

## Results

### Data Processing

**FIGURE 1:**
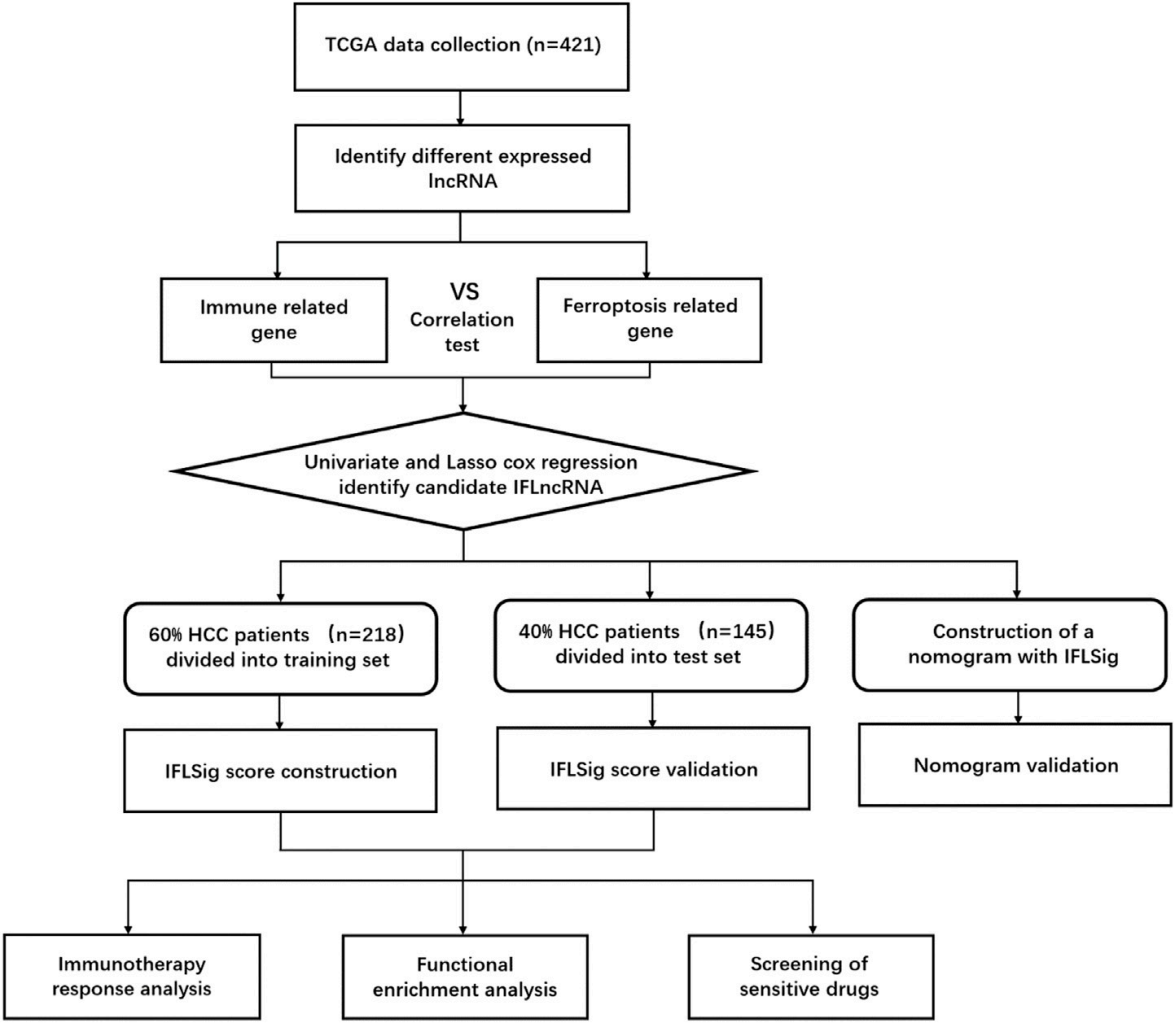
Flow chart of whole design thought.

A total of 15,095 lncRNA RNA-seq were obtained from the TCGA database, and 681 downregulated DEGs were preliminary screened (Figure 1A). We extracted 248 ferroptosis-related genes from the FerrDb database and 453 immune-related genes from the ImmPort database. Pearson correlation analysis was performed between the lncRNAs and ferroptosis-related genes and immune-related genes (with a Correlation coefficient >0.4 and *p* < 0.01). Univariate Cox proportional regression analysis was performed to estimate the prognostic power of lncRNAs. A total of 26 lncRNAs (*p* < 0.05) were identified in the TCGA series. The LASSO Cox regression kept 17 candidate IFLncRNAs after filtration: AC009005.1, AC016773.1, AC090164.2, AC092119.2, AC099850.3, AL021807.1, AL356234.2, AL359510.2, CASC9, DUXAP8, GDNF-AS1, LINC01224, LINC01436, LINC02202, LUCAT1, PTGES2-AS1, and ZFPM2-AS1. LASSO coefficient profiles and optimal penalty parameter lambda are shown in Supplementary Figures S1A,B. The predictive performance of candidate IFLncRNA would be determined in the training cohort to construct a prognostic risk model.

### Construction and Validation of Prognosis Risk Model for HCC

In the training set, the predictive performance of IFLncRNA was determined by multivariate Cox regression. As a result, the total risk score is determined by the following equation:

Risk score(patients)=(0.671∗AC009005.1)+(−2.555∗AC092119.2)+(0.820∗AC099850.3)+(−0.941∗AL356234.2)+(−0.301∗GDNF-AS1)+(0.373∗LINC01224)+(0.218∗LUCAT1)+(0.114∗ZFPM2−AS1)

**FIGURE 2:**
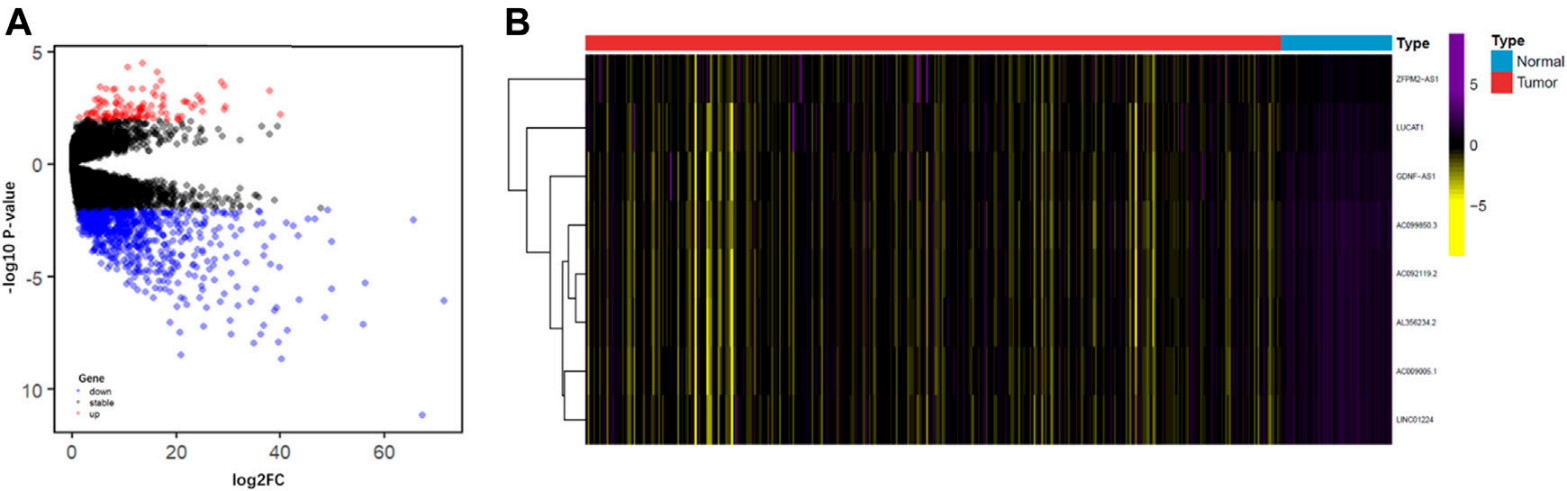
**(A)** Volcano diagram showing the DEGs between cancer and normal samples. The red, black, and blue dots represent the upregulated genes, no difference, and downregulated genes, respectively. **(B)** Heatmap of the expression value of eight IFLncRNAs, differential expression of lncRNA genes; IFLncRNAs, immune- and ferroptosis-related lncRNAs.

**FIGURE 3:**
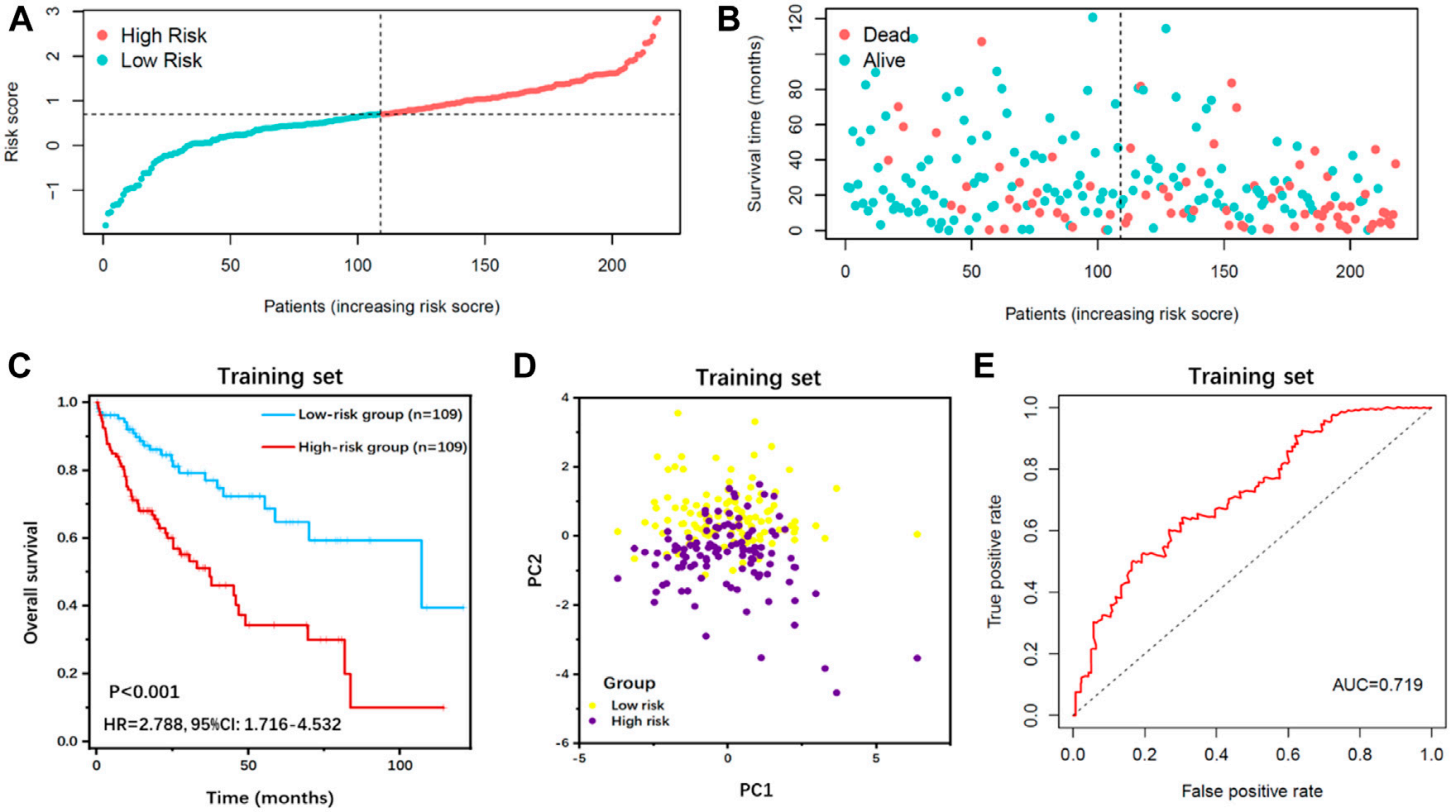
Survival analysis of IFLSig in the training cohort. **(A)** Distribution of IFLSig of HCC cases, which were categorized into two groups based on the median of risk score. **(B)** Distribution of survival time of HCC cases. The positions of the dots reveal the association between the IFLSig and survival time. **(C)** K-M curve for IFLSig relative to the overall survival of the training cohort. **(D)** PCA plot of the training cohort. **(E)** ROC curve of the sensitivity and specificity of survival time on the risk scoreIFLSig, prognostic risk signature of immune- and ferroptosis-related lncRNA; HCC, hepatocellular carcinoma; K-M, Kaplan‐Meier; PCA, principal component analysis; ROC, receiver operating characteristic.

**FIGURE 4:**
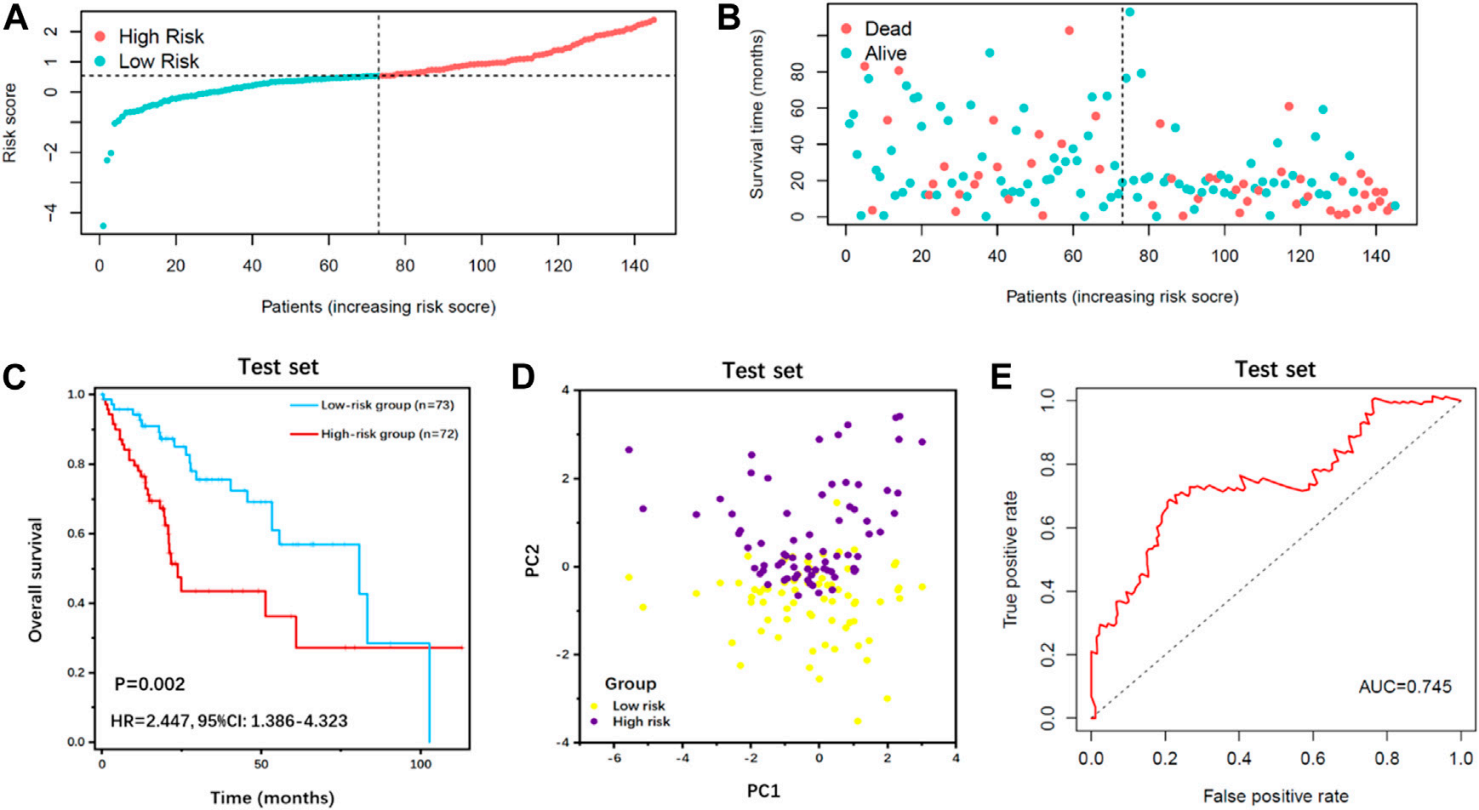
Survival analysis of IFLSig in the test cohort. **(A)** Distribution of IFLSig of HCC cases, which were categorized into two groups based on the median of risk score. **(B)** Distribution of survival time of HCC cases. The positions of the dots reveal the association between the IFLSig and survival time. **(C)** K-M curve for IFLSig relative to the overall survival of the test cohort. **(D)** PCA plot of the test cohort. **(E)** ROC curve of the sensitivity and specificity of survival time on the risk score.

Based on the median value of the risk score, all patients were categorized into the high-risk group or low-risk group in the training cohort (Figures 3A,B) and test cohort (Figures 4A,B). The expression values of eight IFLncRNAs are shown in Figure 2B. In the training cohort, the OS was significantly different between the high-risk group and the low-risk group [Figure 3C, *p* < 0.001, hazard ratio (HR): 2.788, 95% confidence interval (CI): 1.716–4.532]. The same significant difference with the OS was repeated in both groups of the test cohort (Figure 4C, *p* = 0.002, HR: 2.447, 95% CI: 1.386–4.323). As shown in the results, the patients with low risk scores had a better clinical prognosis than the patients with high risk scores, which is a consistent similar outcome for both groups of each cohort. The results of PCA verified the differential expression of IFLncRNA in HCC patients (Figures 3D, 4D). Time-dependent ROC curves of 36 months were applied for the assessment capability of IFLSig with survival time. The area under the curve (AUC) value was 0.719 in the training cohort (Figure 3E) and 0.745 in the test cohort (Figure 4E). All the results suggested that IFLSig might have an accurate predictive ability for OS.

### IFLSig as an Individual Prognostic Variable

To confirm that the IFLSig was an independent prognostic factor of other clinical characteristics, univariate and multivariate Cox regression analyses were performed (Table 1). For the training set, the results of univariate Cox regression analysis (*p* < 0.001, HR: 2.718, 95% CI: 1.995–3.703) and multivariate Cox regression analysis (*p* < 0.001, HR: 2.358, 95% CI: 1.710–3.251) revealed that a high level of IFLSig was significantly associated with shorter survival, and the HR was significantly higher than other factors, whereas patients with a low level of IFLSig were associated with better clinical outcomes. With the same scoring model and risk split point method from the test set, the IFLSig was used to categorize patients into two distinct subgroups. We subjected IFLSig and other clinicopathological features to univariate Cox regression (*p* < 0.001, HR: 2.475, 95% CI: 1.690–3.626) and multivariate Cox regression (*p* < 0.001, HR: 2.590, 95% CI: 1.735–3.865), and IFLSig was still an independent prognostic element for other confounding factors in the test set.

**TABLE 1:** Univariate and multivariate Cox regression analyses in each data set.

Variables	Univariate analysis			Multivariate analysis		
	HR	95％ CI of HR	*p*-value	HR	95％ CI of HR	*p*-value
Training set						
Risk score	2.718	1.995–3.703	<0.001	2.358	1.710–3.251	<0.001
Gender (male/female)	0.822	0.518–1.307	0.408	0.885	0.554–1.412	0.608
Stage (I/II/III/IV)	1.979	1.569–2.497	<0.001	1.706	1.345–2.163	<0.001
Age (<75/≥75)	2.067	1.188–3.597	0.010	1.812	1.036–3.170	0.037
Test set						
Risk score	2.475	1.690–3.626	<0.001	2.590	1.735–3.865	<0.001
Gender (male/female)	0.851	0.481–1.506	0.580	0.882	0.485–1.601	0.679
Stage (I/II/III/IV)	1.254	0.911–1.727	0.164	1.296	0.930–1.807	0.126
Age (<75/≥75)	1.749	0.839–3.644	0.136	1.398	0.658–2.974	0.384

### Functional Enrichment Analysis

**FIGURE 5:**
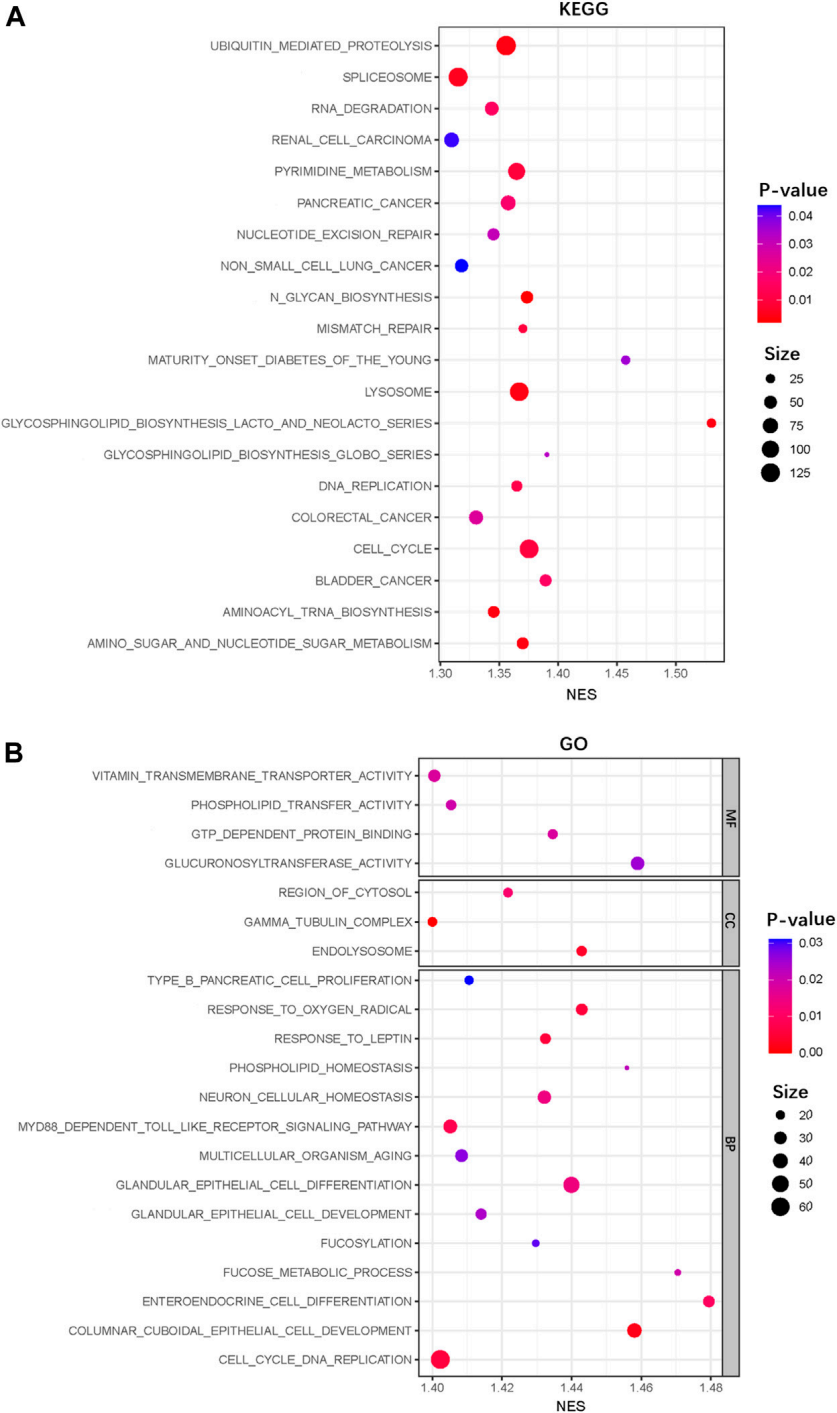
GSEA functional enrichment analysis. **(A)** KEGG enrichment analysis of the IFLSig in the TCGA cohort. **(B)** GO enrichment analysis of the IFLSig in the TCGA cohort. GSEA, Gene Set Enrichment Analysis; KEGG, Kyoto Encyclopedia of Genes and Genomes; TCGA, The Cancer Genome Atlas.

We further performed GO and KEGG pathway enrichment analyses using the GSEA to identify the functional categories and biological pathways of IFLSig. The KEGG pathway analysis revealed main enrichment in cell cycle, spliceosome, lysosome, and ubiquitin-mediated proteolysis (Figure 5A). The results of GO enrichment analyses are shown in Figure 5B. The enriched biological process was mainly involved in the cell cycle DNA replication, glandular epithelial cell differentiation, and columnar cuboidal epithelial cell development. The enriched cellular components were mainly in the region of cytosol, gamma-tubulin complex, and endolysosome. The molecular functions mainly included vitamin transmembrane transporter activity and glucuronosyltransferase activity. We found that most of the enriched functional categories and biological pathways are related to nucleic acid, cell growth, and cell development. This suggested that IFLncRNA may participate in nucleic acid metabolism to regulate cell proliferation, migration, and death to affect the progress of HCC.

### The Potential of IFLSig for Immunotherapy With HCC

**FIGURE 6:**
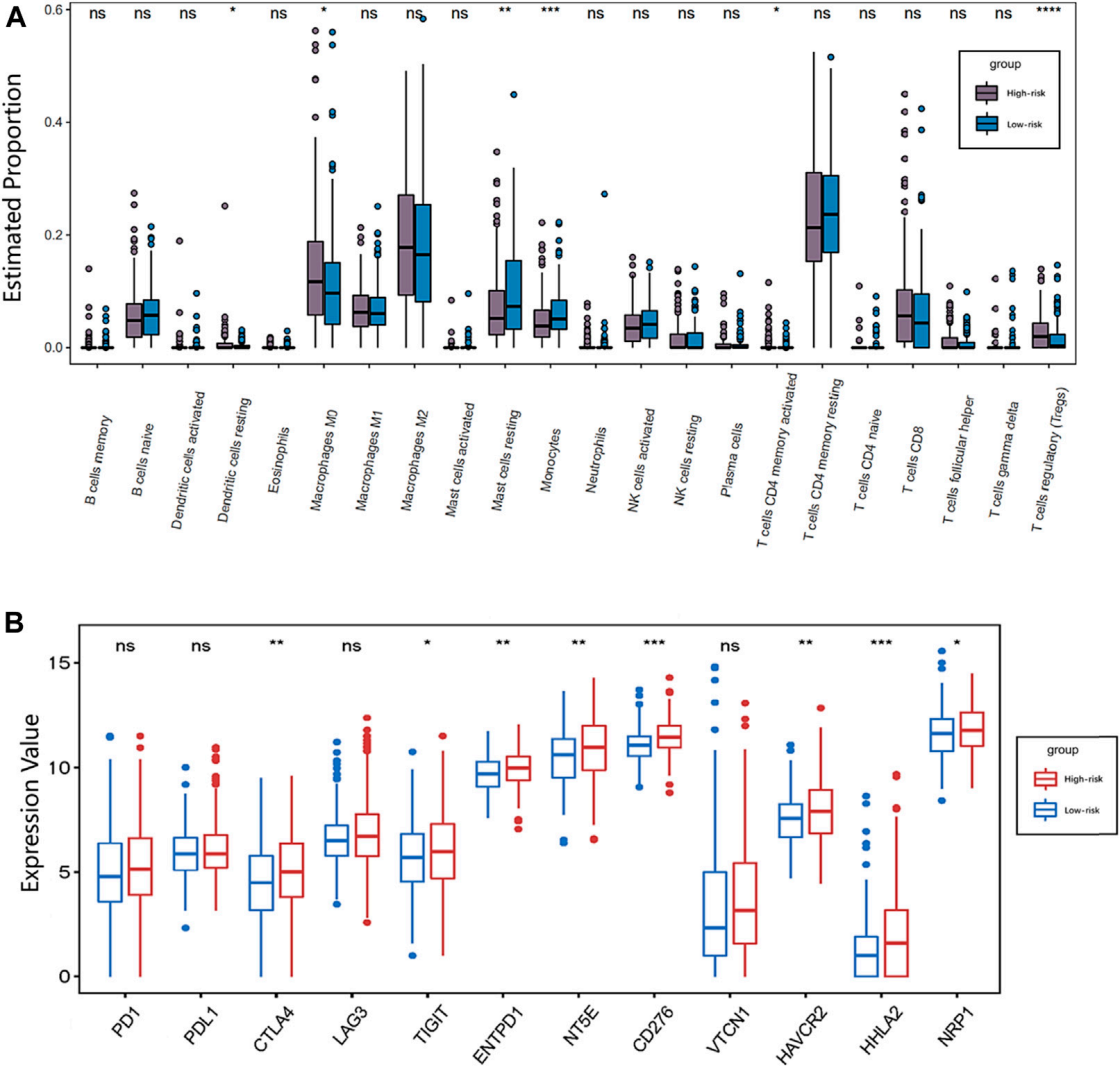
Immunotherapy response analysis. **(A)** Boxplot showing the association between IFLSig and immune cell lines; ANOVA was used as the significance test, *p < 0.05, **p < 0.01, ***p < 0.001, and ****p < 0.0001. **(B)** Boxplot showing the association between IFLSig and immune checkpoints; ANOVA was used as the significance test, *p < 0.05, **p < 0.01, ***p < 0.001, and ****p < 0.0001. GSEA, Gene Set Enrichment Analysis; KEGG, Kyoto Encyclopedia of Genes and Genomes; TCGA, The Cancer Genome Atlas.

To better investigate the complex crosstalk between IFLSig and immune characteristics, we evaluated the immune infiltration profiles of 22 immune infiltration cells in HCC samples by using the CIBERSORT. The proportion of immune profiles and correlation heatmap are shown in Supplementary Figures S2A,B. We further compared the association between immune infiltration cells and IFLSig, and the immune infiltration of most immune cell subtypes was visibly decreased in the high-risk group. Notably, dendritic cells in resting (*p* < 0.05), macrophages in M0 (*p* < 0.05), activated CD4 memory T cells (*p* < 0.05), mast cells in resting (*p* < 0.01), monocytes (*p* < 0.001), and regulatory T cells (Tregs, *p* < 0.0001) increased significantly in HCC patients in the high-risk group (Figure 6A). It is consistent with previous observations linking higher expression of Tregs and macrophages to tumor progression and immunosuppression. Tumor immune infiltration can be adjusted by the immune checkpoints, so we compared the expression value of 12 immune checkpoints and IFLSig. As shown in Figure 6B, the difference in the expressions of CTLA-4 (*p* < 0.01), TIGIT (*p* < 0.05), NRP1 (*p* < 0.05), ENTPD1 (*p* < 0.01), NT5E (*p* < 0.01), HAVCR2 (*p* < 0.01), CD276 (*p* < 0.001), and HHLA2 (*p* < 0.001) was statistically significant. These results indicate that IFLncRNA might be a potential predictive biomarker of response to ICI immunotherapy for HCC.

### Heterogeneous Drug Resistance and Sensitivity With IFLSig

**FIGURE 7:**
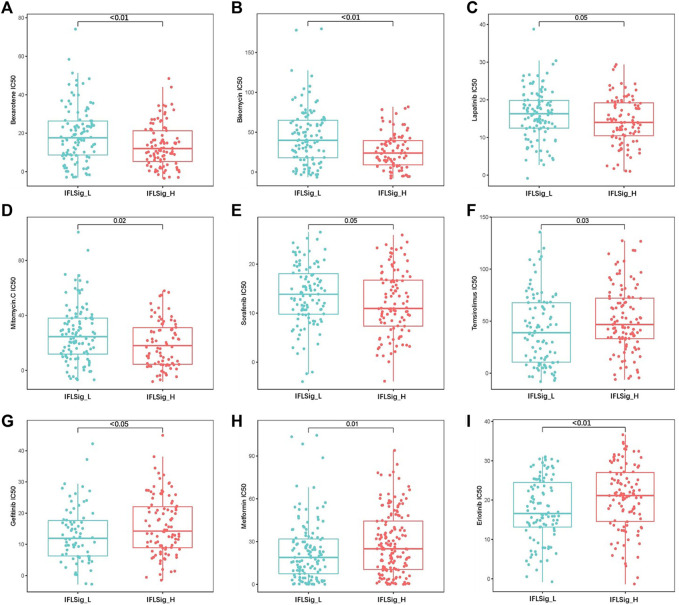
Drug resistance and sensitivity analysis in the low-risk and high-risk groups.

Drug resistance to many chemotherapeutic drugs often occurs during the process of cancer treatment, which leads to poor drug efficacy in liver cancer and worse clinical outcomes. To verify the application of IFLSig in chemotherapy, we compared the half-maximal inhibitory concentration (IC50) and IFLSig. IC50 can help to quantify the therapeutic capacity of a drug to induce cancer apoptosis, which is inversely related to the sensitivity of chemotherapeutics. We evaluated the chemotherapeutic effects of 30 anti-tumor drugs on patients with HCC, and those with *p* > 0.05 were removed. As shown in Figure 7, the IC50 of gefitinib (*p* < 0.05), mitomycin (*p* = 0.02), temsirolimus (*p* = 0.03), and erlotinib (*p* < 0.01) was significantly higher in the high-risk group than in the low-risk group, which means that patients with high IFLSig may not benefit from these drugs. The IC50 of bexarotene (*p* < 0.01), metformin (*p* = 0.01), sorafenib (*p* = 0.05), bleomycin (*p* < 0.01), and lapatinib (*p* = 0.05) was significantly lower in the high-risk group, and therefore, these chemotherapeutic drugs may have a greater effect on patients with high IFLSig. It showed that IFLSig can not only segregate individuals into different risk groups but can also assist in selecting chemotherapeutic drug pools based on the sensitivity values corresponding to the HCC patients under clinical observation.

### Identification and Validation of a Nomogram

**FIGURE 8:**
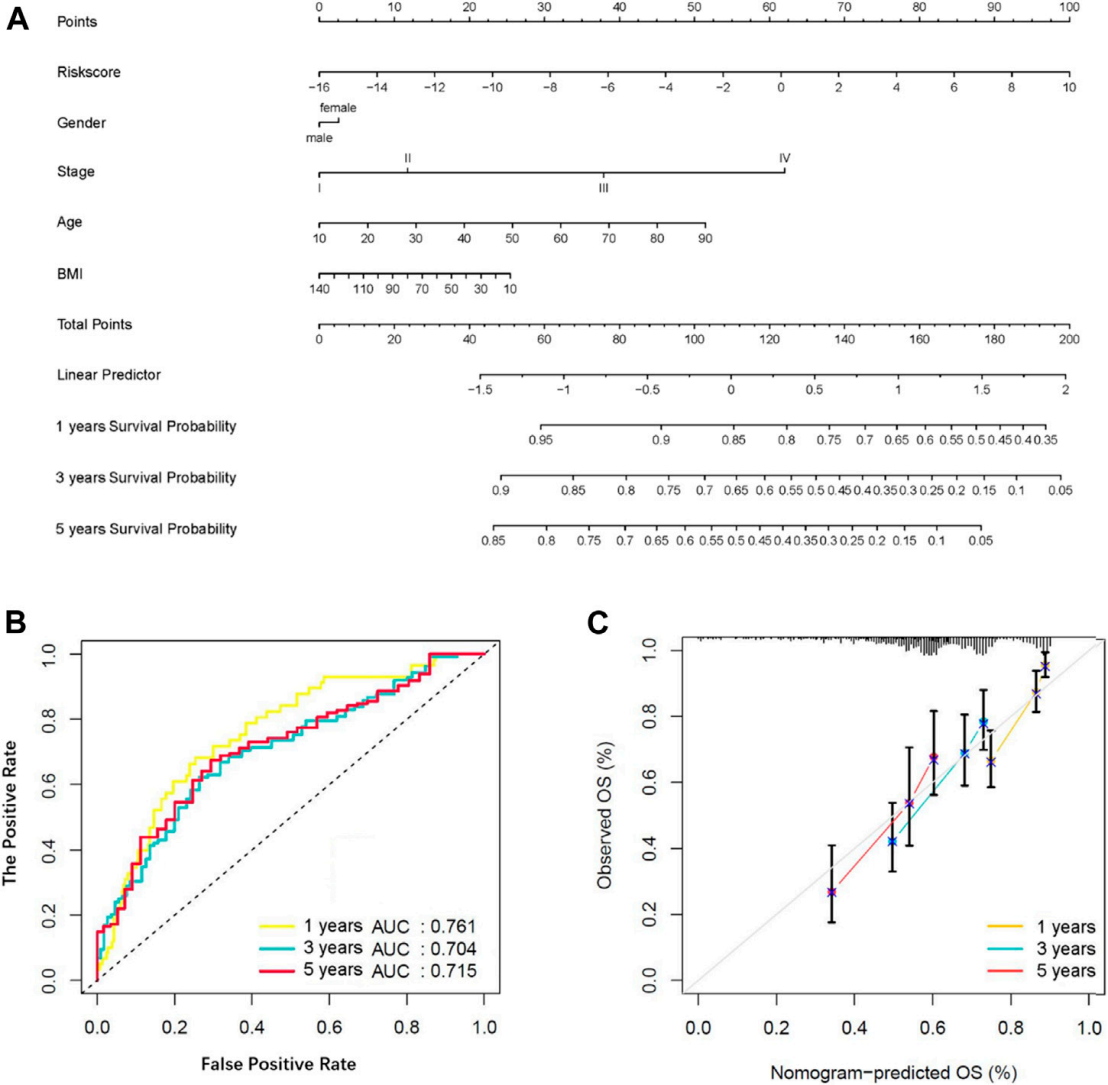
Construction and verification of a nomogram. **(A)** Survival nomogram based on total TCGA cohort. **(B)** The ROC curve compared the prognostic power of the nomogram at 1, 3, and 5 years in the TCGA cohort. **(C)** The calibration curve for predicting HCC patient survival at 1, 3, and 5 years in the TCGA cohort.

The nomogram is a reliable tool to estimate individualized risk score in cancer patients. In this study, we constructed a nomogram based on the entire TCGA set by using the multivariate Cox regression analysis of the IFLSig and other clinicopathological covariables (Figure 8A), and it was internally validated in the training cohort and test cohort by using the *C*-index. The *C*-index of the nomogram was 0.675 (95% CI: 0.623–0.727) in the entire TCGA set, 0.7108 (95% CI: 0.653–0.769) in the training cohort, and 0.633 (95% CI: 0.539–0.726) in the test cohort. The AUC of the nomogram were 0.761, 0.704, and 0.715 for 1, 3, and 5 years, respectively, showing that the nomogram had a good level of specificity and sensitivity for OS (Figure 8B). The calibration plot is in agreement with the diagonal line, and it confirmed the predictive value of the prognostic nomogram for OS at 1, 3, and 5 years (Figure 8C). All the results demonstrated that the nomogram constructed by IFLSig had good prognostic power in HCC patients.

## Discussion

Recently, lncRNA has been suggested to play an important role in the oncogenesis and progression of HCC (Ding et al., 2018; Guo et al., 2020). Multiple lncRNAs have been suggested to possess aberrant expression and participate in cancerous phenotypes through their binding with hereditary substances, encoding proteins or other small molecule peptides (Wong et al., 2018; Pan et al., 2019; Peng et al., 2020). Simultaneously, clinical management places emphasis on the importance of early and effective disease detection and prediction of prognosis. This requires finding the right types of components, biomarkers, and detection methods that could be applied to tumor detection and treatment. Historically, some prognostic models have been constructed for clinical practice, but the accuracy, safety, and effectiveness should be evaluated over a long period of time. Therefore, we developed a lncRNA prognostic risk model combined with ferroptosis and immunity to provide new insights into HCC pathogenesis and an effective tool for predicting the treatment efficacy of HCC, which may help bring about additional therapeutic and prognostic benefits. Research suggests that immunity is suppressed in the process of hepatocarcinogenesis, so we selected downregulated immune genes to develop the prognostic model linked to previous studies (Sun et al., 2020; Zhou et al., 2021). In this study, cancer samples were allocated into IFLSig high-risk group and low-risk group. IFLSig was the dominant factor in the prognostic risk model and nomogram. Our results achieved a satisfactory association with clinical outcomes, which suggested that IFLSig is an excellent prediction risk factor. We analyzed the resistance and sensitivity of chemotherapeutic drugs to verify the predictive potential of IFLSig to determine therapeutic efficacy. In summary, we have developed an IFLSig index that was closely associated with the prognosis of HCC, and it combines immunological characteristics to better estimate OS and can predict clinical therapy response for HCC. Our IFLSig model would hopefully provide new insights into HCC pathogenesis and novel tools to improve prognosis prediction and determination of treatment efficacy in patients with HCC.

IFLncRNA has been confirmed to exert a pivotal function in the proliferation, differentiation, invasion, and metastasis of liver tumors through different pathways in tumors progression and pathogenesis. Presently, it has been confirmed that ZFPM2-AS1, LINC01224, and LUCAT1 possess differing expression levels in liver cancer tissues and are involved in multiple processes promoting hepatocarcinogenesis, including enhancing proliferation of tumor cells, anti-apoptosis, improving the migration ability of cancer cells, and strengthening the invasiveness of cancer cells (He et al., 2020; Liu et al., 2020). LINC01224 can specifically upregulate the anti-apoptotic protein CHEK1 to influence the cancer cells (Gong et al., 2020). LUCAT1 can activate the metalloproteinase protein, regulating the expression of DLC1, and enhance the malignant phenotype of hepatoma cells (Lou et al., 2019; Wu et al., 2020). GDNF-AS1 is a natural antisense transcript that can increase the expression of site-specific gene GDNF after its knockout (Modarresi et al., 2012). GDNF can significantly activate hepatic stellate fibroblasts and promote liver fibrosis, participate in the microenvironment of liver cancer, affect the metabolism of extracellular matrix, and is closely related to the occurrence and progression of primary liver cancer (Tao et al., 2019; Barry et al., 2020).

In the tumor microenvironment, cancer cells and immune cells exert a great number of chemokines and cytokines to regulate tumor pathogenesis and progression. In this study, we found that the increase in regulatory T cells was particularly significant. The robust immunosuppressive microenvironment in cancer represents a crucial challenge for cancer treatment. Tregs and tumor-associated macrophages can directly decrease the proliferation of T cells in the immune microenvironment (Peterson et al., 2018; Ohue and Nishikawa, 2019). It can also influence tumor aggressiveness by affecting lactate metabolism (Wang et al., 2020). Our study suggested that IFLncRNA of HCC patients might be related to immune escape. However, the mechanism between IFLncRNA and Tregs remains obscure, and further research will be required to resolve this question. We further evaluated the expression level of these immunosuppressive checkpoint inhibitors, and IFLSig showed great relativity with CTLA-4, TIGIT, NRP1, ENTPD1, NT5E, HAVCR2, CD276, and HHLA2. In addition, the immunotherapy of anti-CTLA4 has been widely used in the treatment of liver cancer (Agdashian et al., 2019; Lai et al., 2021; Martini et al., 2021). Based on GSEA, we found that IFLSig was enriched in ubiquitin-mediated proteolysis, RNA degradation, lysosome, and cell cycle, which can strongly influence the metabolism of PD1 and PDL1. Although there is no significant difference between IFLSig and PD1 or PDL1, IFLSig may influence PD1 and PDL1 by affecting the above-listed molecular mechanisms (Burr et al., 2017; Gou et al., 2020; Kalbasi and Ribas, 2020; Zhong et al., 2021). In summary, we believe the results show that IFLSig has the potential in predicting the effectiveness of immunotherapy.

There are some shortcomings in this study. The prediction capacity of the motioned prognostic signature failed to be authenticated in the GEO cohort and ICGC cohort because none of the cases included the expression array of IFLncRNA. We were also concerned about whether the plain correlation test can accurately calculate the correlation between lncRNA, ferroptosis, and immunity. It should be noted that this study is a retrospective analysis based on bioinformatics data without yet the validation of a prospective analysis. Therefore, more experimental and clinical data are needed for verification.

## Conclusion

In conclusion, we developed an IFLSig prognostic index based on eight IFLncRNAs, which are related to the clinical outcomes of HCC, by performing many bioinformatics statistical analyses. The IFLSig can be considered an independent prognostic signature that may be able to estimate the OS and clinical therapy response in HCC patients. This study provides a new understanding of IFLncRNA in the development and progression of HCC.

## Data Availability

The original contributions presented in the study are included in the article/Supplementary Material, further inquiries can be directed to the corresponding author.
