# Supplementary material for: Computational Identification of Immune- and Ferroptosis-Related LncRNA Signature for Prognosis of Hepatocellular Carcinoma
Source: Front Mol Biosci. 2021 Nov 25;8:759173. doi: 10.3389/fmolb.2021.759173 (PMC8655914; doi:10.3389/fmolb.2021.759173)
Supplement: Supplementary file 4 [file DataSheet1.docx]

Supplementary Material

## Supplementary Figures

**Supplementary Figure 1.** LASSO Cox regression analysis. (A) LASSO coefficient profiles. (B) The optimal penalty parameter lambda of LASSO Cox regression.


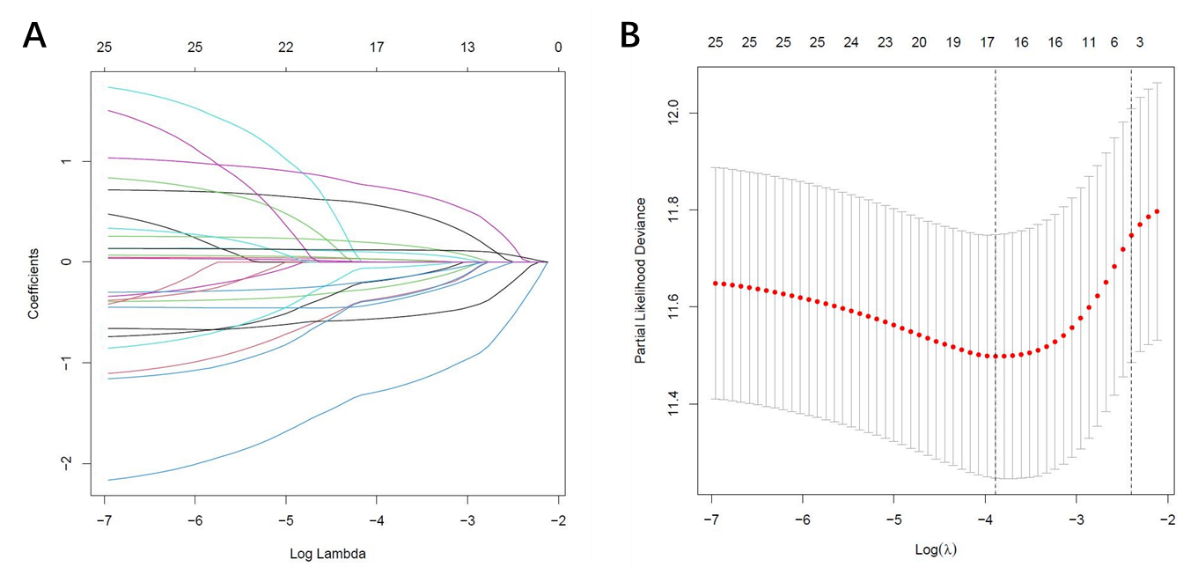


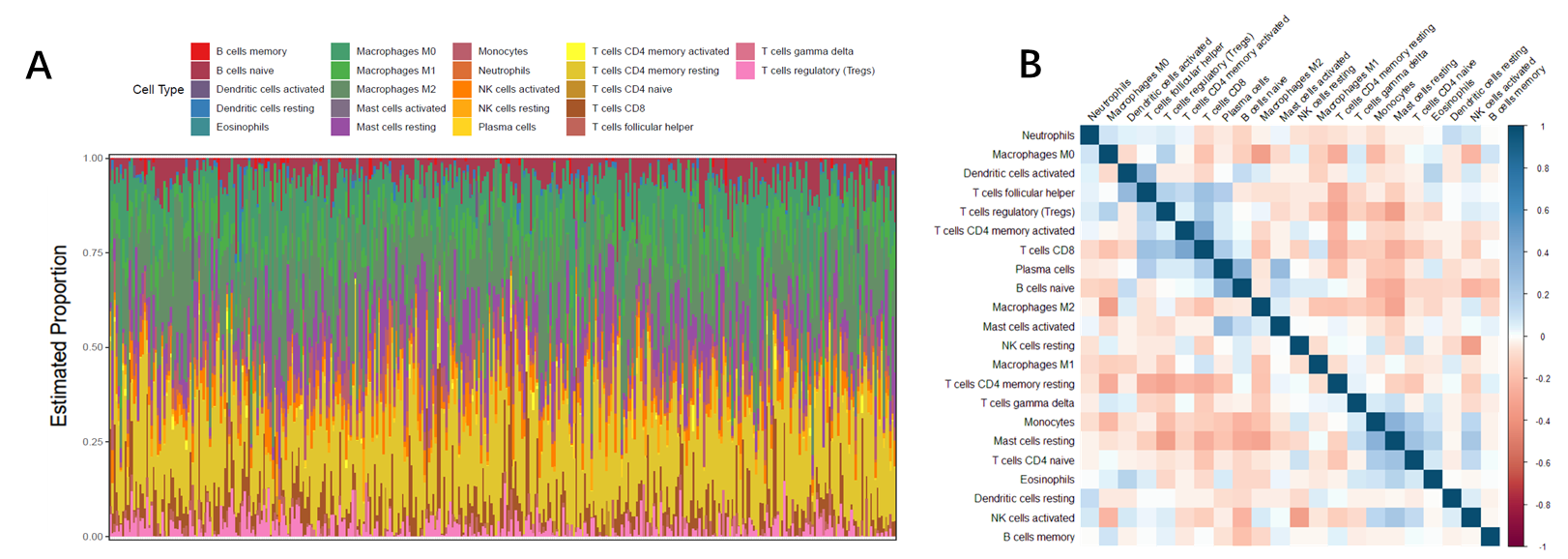
**Supplementary Figure 2.** Supplementary Figure 2| Immune infiltration of HCC patients in TCGA cohort. (A) A bar plot showing the proportion of 22 immune cell lines in HCC patients. (B) Correlation heat map displaying the correlation between each of 22 different immune cell lines, the shade of each color block shown the corresponding correlation value between two cells.
